# G protein pathway suppressor 2 (GPS2) acts as a tumor suppressor in liposarcoma

**DOI:** 10.1007/s13277-016-5220-x

**Published:** 2016-07-26

**Authors:** Xiao-Dong Huang, Feng-Jun Xiao, Shao-Xia Wang, Rong-Hua Yin, Can-Rong Lu, Qing-Fang Li, Na Liu, Ying zhang, Li-Sheng Wang, Pei-Yu Li

**Affiliations:** 1 Department of General Surgery, Chinese PLA General Hospital, 28 Fuxing Road, Beijing, 100853 China; 2 Department of Experimental Hematology, Beijing Institute of Radiation Medicine, 27 Taiping Road, Beijing, 100850 China

**Keywords:** GPS2, Liposarcoma, DDLPS, WDLPS

## Abstract

Liposarcoma(LPS) is the most common type of soft tissue sarcoma accounting for 20 % of all adult sarcomas. However, the molecular pathogenesis of this malignancy is still poorly understood. Here, we showed that GPS2 expression was downregulated in LPS and correlated with the prognosis of this disease. In vitro study showed that knockdown of GPS2 resulted in enhanced proliferation and migration of LPS cell line SW872, without significant influence of cell death. Conclusively, our results suggest that GPS2 may act as a tumor suppressor in LPS and serve as a potential prognosis marker for this disease.

## Introduction

Liposarcoma (LPS) is the most common subtype of soft tissue sarcoma, accounting for 24 % of extremity and 45 % of retroperitoneal soft tissue sarcomas [1]. According to the World Health Organization (WHO) classification guideline, LPSs are divided to three subtypes: well-/dedifferentiated (WD/DD) LPS, myxoid/round-cell (MRC) LPS, and pleomorphic LPS (PLS) [2]. Among them, (WD/DD) histologies are the most frequent types existed in 40–50 % of LPS cases [3]. WDLPS are low-grade tumors with a more indolent course, while DDLPS are high-grade, aggressive tumors with a 15–20 % risk of distant metastasis and approximately 30 % 5-year survival rate [4]. Until now, surgery is the only curative therapeutic strategy for localized disease, as these tumors are largely resistant to conventional cytotoxic chemotherapy and radiotherapy as well [5, 6]. However, there are still 58 to 80 % of patients with DDLPS of the retroperitoneum will succumb to locally recurrence or metastasis within 5 years [7]. Thus, it is of great urgency to develop effective targeted therapies to improve outcomes of patients with liposarcoma. Nevertheless, it has been hampered by a lack of understanding of the pathogenesis of this disease.

In the past decade, new approaches, including gene expression profiling, DNA copy number profiling, whole-exome sequencing, miRNA profiling, and RNA sequencing, have successfully used to expand our understanding of LPS pathogenesis [7–10]. Amplification of chromosome 12q14-15 was the best-studied molecular aberration in LPS, which results in amplification of the MDM2 (located at 12q15) and CDK4 gene in over 90 % of WD/DD LPS cases [11–14]. This amplification was reported to cause decreased apoptosis and increased cell proliferation, which were thought to be resulted from dysregulation of the p53 pathway and the Rb-E2F cell cycle checkpoint [4, 14]. Multiple mechanisms including genetic amplifications, overexpressions of receptor tyrosine kinases (RTKs), chromosome translocation, deregulation of PI3K/Akt pathway, dysregulation of some miRNAs, and deregulation of epigenetics are reported to be associated with the development of LPS [4].

G protein pathway suppressor 2 (GPS2), located at 17p13.1, was originally identified as a suppressor of Ras activation in the yeast [15]. It was later shown to be involved in many physiological and pathological processes, including proliferation, apoptosis, DNA repair, brain development, and metabolism [16–20]. Dysregulation of GPS2 has been reported to be associated with tumorgenesis of glioblastoma multiforme and undifferentiated spindle cell sarcoma (UDS) [21]. However, the role of GPS2 in tumorgenesis remains largely unknown.

In view of that GPS2 is downregulated in the adipose tissue of obese individuals and that it plays a critical role in the regulation of adipose tissues [22], we attempt to explore the potential role of GPS2 in LPS, the most common subtypes of soft tissue sarcoma. Our results indicated that GPS2 act as a tumor suppressor in LPS and it may serve as a prognosis marker in this disease.

## Materials and methods

### Cell culture

The human SW872 cell line was obtained from the Department of Public Health of Harbin Medical University. The cells were grown in DMEM supplemented with 10 % FBS, penicillin (100 U/ml), at 37 °C in 5 % CO_2_.

### Lentivirus transduction

The pSicoR-GFP vectors carrying the GPS2-shRNA-1 sequence GAGGAGACCAAGGAACAAA or GPS2-shRNA-2 sequence GGAAGAGAGAATGTCATTA were used as in this study. The lentiviruses were packaged by cotransfected lentivirus vector with psPAX2 and pMD2.G into 293T cells. The gene transduction efficiency of lentiviral vectors, indicated by GFP expression, was detected by flow cytometry (Becton Dickinson, Mountain View, CA). SW872 cells were plated in six-well plate at a density of 2 × 10^5^ per well, and then were infected by lentiviral vectors at multiplicity of infection (MOI) of 10.

### Wound-healing and migration assays

To analyze wound-healing ability of SW872 cells, 2 × 10^5^ cells were seeded in six-well plates with DMEM supplemented with 10 % FBS. After 24 h, the cell monolayer was wounded using a plastic pipette tip. The cells were then rinsed with PBS and cultured with serum-free DMEM for 24 h. The wound closure was observed and photographed under a microscope.

The migration assay was performed using Transwell inserts (Costar, NY, USA; pore size, 8.0 μm) in 24-well cell plates. SW872 cells transduced with 10 MOI lentivirus for 48 h was assayed. Approximately 1 × 10^5^ cells in 100 μl serum-free medium were placed in the upper chamber, and 600 μl of the same medium containing 10 % FBS was placed in the lower chamber. The plates were incubated for 5 h at 37 °C in 5 % CO_2_; then, cells were washed with 600 μl PBS for twice and fixed in 2 % paraformaldehyde for 20 min. After that, nonmigrating cells in the upper chambers were removed with a cotton swab; migrated cells on the lower surface of the porous membrane were remained. Migrated cells were stained with crystal violet for 10 min and photographed under an optical microscope. Five random fields (×100) were chosen in each well, and the cell number was quantified manually. The average number of migrated cells of five fields was compared among two groups.

### RNA extraction and quantitative RT-PCR

Total RNA was extracted with TRIzol reagent (Invitrogen Life Technologies, Carlsbad, CA, USA), and cDNA was synthesized using Revert Aid™ First Strand cDNA Synthesis Kit (Thermo Scientific, Wilmington, DE) according to the technical manual. The mRNA expression of target genes was quantified using 7500 Fast Real-Time PCR System (Applied Biosystems, Foster City, CA) and SYBR® Premix Ex Taq™ II (Takara, Japan). Their expression levels were normalized by β-actin. The PCR primers were designed and synthesized in AUGCT Biological Technology Co., Ltd., Beijing, China. The primers are listed in Table 1.

**Table 1:** Primers used in this study

Gene	Primers
β-Actin	Sense 5′GACATGCCGCCTGGAGAAAC3′
	Anti-sense 5′AGCCCAGGATGCCCTTTAGT3′
Gps2	Sense 5′ACATTATGATGGAGCGGGAG 3′
	Anti-sense 5′GCTGGTGCTTCTCTTCCTGTAG 3′
C/EBPα	Sense 5′GTCCAAACCAACCgCACAT3′
	Anti-sense 5′GAAACAACCCCGTAGGAACAT3′
LPL	Sense 5′ACTGCCACTTCAACCACAGC3′
	Anti-sense 5′AATACTTCGACCAGGCGACC3′
Pref1	Sense 5′GCGCGGGACTCCAGCCCTAAGT3′
	Anti-sense 5′GCGGTGCAGGGGCTGCTCCGGG3′
PPARγ1	Sense 5′GTTGATTTCTCCAGCATTTCCA3′
	Anti-sense 5′GGCTCTTCGTGAGGTTTGTTG3′
PPARγ2	Sense 5′GAACTTAACTGCAGGACCTGCG3′
	Anti-sense 5′AACTGATGCTGACGAGTGCCT3′

### Protein extraction and Western blot

Cells were washed twice with 1× PBS, and protein was extracted from SW872 transduced with lentivirus. Total cell protein concentrations were confirmed using BCA protein assay kit. Then, 40 μg of the sample was separated by sodium dodecylsulfate–polyacrylamide gel electrophoresis (SDS-PAGE) and transferred to PVDF membranes. After blocking with 5 % nonfat milk in Tris-buffered saline containing 0.1 % Tween 20 (TBST), the membranes were incubated at room temperature for 5 h with primary antibody (mouse anti-GPS2 (1:1000 dilution, made by our lab); mouse anti-GAPDH (1:1000 dilution, ASGB-BIO, China); mouse anti-Cyclin D1 (1:1000 dilution, CST, USA), mouse anti-PPARγ (1:1000 dilution, CST, USA)). The membranes were then washed three times with TBST and incubated with the secondary antibody at room temperature for 50 min in 5 % milk in TBST. After five washes with TBST, the membranes were visualized with enhanced chemiluminescence detection kit (ECL, Amersham Pharmacia). GAPDH functioned as an internal control. Tanon 5200 imaging system (Tanon Company Ltd., Shanghai, China) was used to acquire the blots.

### DYE670 staining assay

The GPS2-shRNA and ctrl-shRNA transduced cells were washed two times with PBS to remove serum, then resuspended at 1.0 × 10^6^/ml in PBS (prewarmed to room temperature). The cell suspension was mixed with a solution of Cell Proliferation Dye eFluor® 670 and incubated for 10 min at 37 °C in the dark, then labeling was stopped by adding 4–5 volumes of cold complete media (containing 10 % serum) and incubated on ice for 5 min. After washed for three times with complete media, the cells were cultured at 5 % serum medium at 37 °C in 5 % CO_2_ for 0, 48, 72, and 96 h, respectively. These cells were collected and fixed in 2 % paraformaldehyde at 4 °C for flow cytometry assay. Flow cytometric analysis was conducted on a FACSCalibur can using the Cell Quest software package (Becton Dickinson, CA).

### BrdU staining assay

For the BrdU assay, cells were seeded in six-well plates and cultured in DMEM supplemented with 10 % FBS or control media for 24 h and then 10 μM BrdU (BrdU assay kit, Kai Ji, Nanjing, China) was added. After 8 h, cells were rinsed twice with PBS and then were incubated in working liquid at 37 °C for 30 min. At last, cells were suspended in 200 μl of staining buffer with 5 μl of a PE-BrdU antibody for 30 min at 4 °C without light and were imaged by an IX70 inverted microscope (Olympus, Hamburg, Germany). Experiments were performed in triplicate and tracks of at least ten cells from three positions were analyzed in each well for different conditions.

### Apoptosis assays

Annexin V/phosphoinositide (PI) apoptosis detection kit was used to detect externalization of phosphatidylserine according to the manufacturer’s instructions (BD Pharmingen). In brief, 1 × 10^6^ SW872 cells were washed twice with PBS and stained with 5 μl of Annexin V-FITC for 15 min and 10 μl of PI in binding buffer (10 mM Hepes, pH 7.4, 140 mM NaOH, and 2.5 mM CaCl_2_) for 15 min at 4 °C. Flow cytometric analysis was conducted on a FACSCalibur using the Cell Quest software package (Becton Dickinson). Apoptotic cells were defined as Annexin V+/PI−.

### Cell cycle analysis

For flow cytometry measurements of the cell cycle, 48-h posttransfection cells were collected, centrifuged at 300×*g* for 5 min, and fixed overnight in 70 % cold ethanol at −20 °C. After washing twice with PBS, the cells were resuspended in 500 μl of fresh PBS containing 50 μl of 2 mg/ml RNaseA and 10 μl of 1 mg/mL PI (Sigma). Cells were incubated for 15 min at 37 °C. The cells were then analyzed immediately using a FACSCalibur using the Modfit software (Becton Dickinson) at 30,000 events/sample.

### Glucose concentration test

The concentrations of glucose in culture medium were determined by the glucose oxidase method in North of Beijing Institute of Biotechnology Co., Ltd.

### Tumor and normal tissue samples

For the human tissue samples, a total of 50 fresh tumor samples and corresponding normal samples were obtained from patients in the Chinese PLA General Hospital during surgery. Clinical data about 50 patients with retroperitoneal liposarcoma managed with surgery in our hospital from January 2003 to December 2014 were retrospectively analyzed, and all patients were followed up. Informed consent was obtained from all patients prior to tissue collection. No patients had received radiation therapy or chemotherapy prior to surgery. The clinicopathological information of patients, including age, gender, histological type, and median follow-up was obtained from patient records and is summarized in Table 2.

**Table 2:** The clinicopathological information of LPS patients

Characteristics	Value
Age	
Mean ± SD	51.79167 ± 12.7824 years
Range	26–85 years
Gender	
Male	28 (56 %)
Female	22 (44 %)
Histologic subtype	
WD	27 (54 %)
DD	23 (46 %)
Median follow-up	28 months

### Immunohistochemistry

Commercially available monoclonal antibodies against CDK4 (Clone DCS-31, 1:50, Invitrogen), MDM2 (Clone IF-2,1:75, Zymed Laboratories), and GPS2 (1:100)were used. Anti-GPS2 antibodies for Western blotting were made by our lab (1:100). All WDLPS and DDLPS cases were stained for CDK4, MDM2, and GPS2 by immunohistochemistry. Sections were deparaffinized and rehydrated in graded alcohol. For heat-induced epitoperetrieval (HIER), the sections were subjected to DIVA retrieval buffer (pH 6.0) in a pressure cooker (Biocare Medical) at 98 °C for 60 min. The sections were then brought to an automated stainer (DAKO) following the vendor’s protocol. EnVisionPlus and peroxidase detection methods were used and counterstained in 50 % Mayer’s hematoxylin for 1 min. Nuclear CDK4 immunoreactivity, nuclear and cytoplasmicMDM2 immunoreactivity, and nuclear GPS2 immunoreactivity were assessed. Those cases with more than 1 % of tumor cells showing positive staining were assessed as positive. The staining was graded as + (1–5 % tumor cells positive),++ (5–24 % tumor cells positive), and +++ (>25 % tumor cells positive). The immunohistochemistry staining results of patients were shown as heat maps.

### Adipogenic differentiation

The cells were cultured in adipogenic differentiation media (Lonza, USA)for 9 days. Adipogenic differentiation was characterized with Oil Red O staining. The cells were fixed in 4 % paraformaldehyde, washed, and treated with 60 % ethanol for 5 min. Oil Red O stain was added for 10 min, and cells were imaged for fat droplet formation using the Olympus microscope (Hamburg, Germany).

### Statistical analysis

All results are representative of three independent experiments. Values were presented as mean ± SD. All data were analyzed by using SPSS19.0 and GraphPad Prism 5. Differences between the experimental groups and control groups were assessed by the Student’s *t* test (two-tailed). The results of immunohistochemistry were analyzed by Mann–Whitney test. The Kaplan–Meier method was used to describe and evaluate the survival rate, and the log-rank test was used to compare the survival curves. Data with *P* < 0.05 were considered statistically significant.

## Results

### GPS2 expression and its correlation to prognosis of LPS

**Fig. 1:**
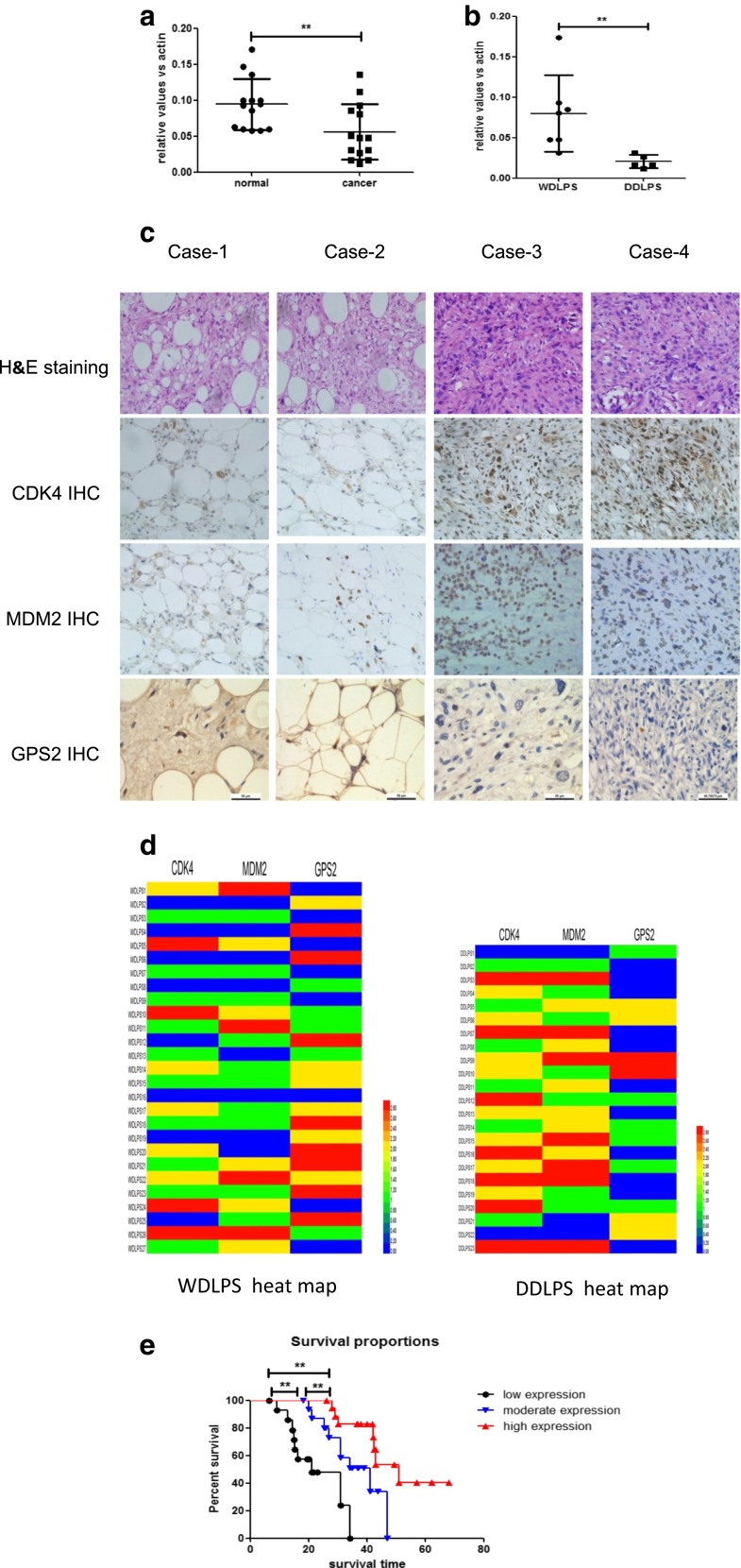
GPS2 expression in LPS. **a** Relative GPS2 expression in 14 LPS samples and their paired normal adipose tissues. **b** Relative GPS2 expression in WDLPS and DDLPS in the LPS samples. **c** IHC staining of CDK4, MDM2, and GPS2 proteins in WDLPS and DDLPS. H&E staining (×200) shows the morphologic characters of four cases of WDLPS and DDLPS. IHC staining of CDK4, MDM2, and GPS2 protein showed variable expression patterns of WDLPS and DDLPS. **d** The heat map of immunohistochemistry staining results of CDK4, MDM2, and GPS2 in samples from LPS patients. **e** Kaplan–Meier survival analysis showed the different prognosis of different expression of GPS2. Data for gene copy number are presented as ratio to β-actin. Significance, **P values < 0.01

To explore the potential role of GPS2 in liposarcomas, we collected 14 LPS samples and their paired normal adipose tissues. qPCR analysis showed that the mRNA levels of GPS2 were significantly lower in tumor samples than their normal control (Fig. 1a). Further clinicopathologic analysis revealed that those samples containing seven WDLPS, five DDLPS, and two other subtypes of LPS. We next compared the GPS2 expression in WD and DD LPS. GPS2 mRNA levels were lower in DDLPS than that in WDLPS (Fig. 1b). To further confirm these results, we collected 50 samples with 27 WDLPS and 23 DDLPS. Immunohistochemical analysis showed that 74.07 % of WDLPS were GPS2 positive whereas only 52.17 % of DDLPS were GPS2 positive (Fig. 1c, d). We analyzed the MDM2 and CDK4 expression synchronously. In accordance with previous reports, MDM2 was positive in 76.00 % cases and the percentage of CDK4 positive was 80.00 % (Fig. 1c, d). The preventative photographs were exhibited as Fig. 1c. Collectively, these results suggested that GPS2 expression was downregulated in LPS and especially in high-grade tumors, implicating GPS2 may has a role in the genesis of these tumors.

It is suggested that GPS2 downregualtion may associate with the malignant degrees of LPS. This assumption conducted us to examine the correlation of GPS2 expression levels to predict clinical outcomes. Twenty-three patients with DDLPS and 27 individuals with WDLPS were enrolled in this prospective study. The general information of these patients is listed in Table 2. Kaplan–Meier survival analysis showed that patients whose tumors had lower GPS2 expression levels had a worse prognosis (Fig. 1e). These data indicated GPS2 may serve as a prognosis marker of these diseases (Table 3).

**Table 3:** The immunohistochemistry staining results of samples from LPS patients

Items	Percentage	CDK4	MDM2	GPS2
DD (*n* = 23)	−	8.70 % (2/23)	13.04 % (3/23)	47.83 % (11/23)
	+	26.09 % (6/23)	30.43 % (7/23)	26.09 % (6/23)
	++	30.43 % (7/23)	26.09 % (6/23)	17.39 % (4/23)
	+++	34.78 % (8/23)	30.43 % (7/23)	8.70 % (2/23)
	Sum of positive	91.30 % (21/23)	86.96 % (20/23)	52.17 % (12/23)
WD (*n* = 27)	−	29.63 % (8/27)	33.33 % (9/27)	25.93 % (7/27)
	+	37.04 % (10/27)	37.04 % (10/27)	22.22 % (6/27)
	++	18.52 % (5/27)	18.52 % (5/27)	22.22 % (6/27)
	+++	14.81 % (4/27)	11.11 % (3/27)	29.63 % (8/27)
	Sum of positive	70.37 % (19/27)	66.67 % (18/27)	74.07 % (20/27)
Total positive		80.00 % (40/50)	76.00 % (38/50)	64.00 % (32/50)
*P*		0.015	0.015	0.025

### Enhanced proliferation in GPS2 knocked-down SW872 cells

**Fig. 2:**
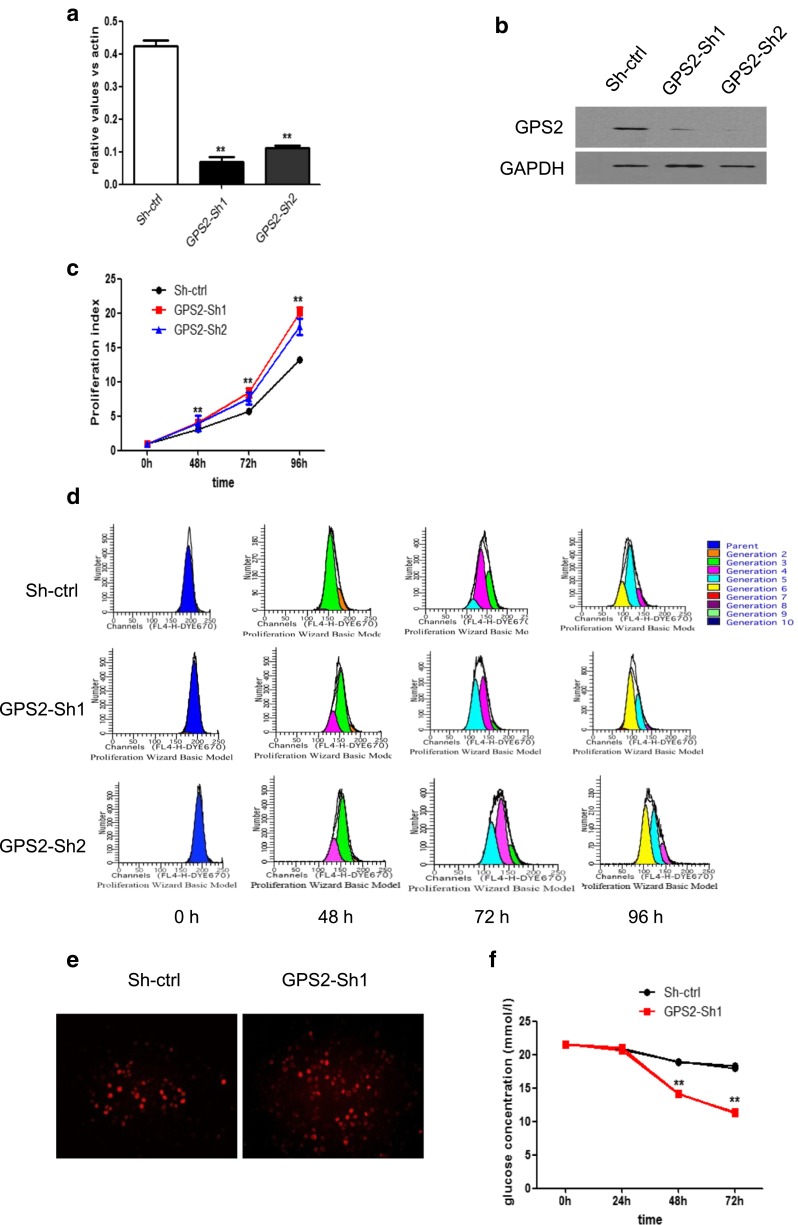
GPS2 knockdown enhances proliferation of SW872 cells. **a** SW872 cells transfected with sh-ctrl, GPS2-sh1, and GPS2-sh2 were subjected to q-PCR analysis. GPS2 mRNA levels were normalized to β-actin. **b** GPS2 protein levels were determined by Western blot, GAPDH as a loading control. **c** SW872 cells transfected with sh-ctrl, GPS2-sh1, and GPS2-sh2 were labeled with Dye670 and cultured for 4 days. Cell division was evaluated on the basis of reduction of Dye670 fluorescence intensity. A proliferation index (PI) was calculated using ModFit software. **d** The representative plot of Dye670-labeled cells for proliferation index assay. **e** The cells infected with sh-ctrl or GPS2-sh1 were photographed using BrdU staining. **f** Glucose concentrations in the medium of cells infected with sh-ctrl or GPS2-sh1 were detected from day 0 to day 4. Cumulative results represent the mean ± SEM. Significance, **P < 0.01

To explore the roles of GPS2 in regulation of biological characteristics of liposarcoma cells, we checked the effect of loss of GPS2 on proliferation, migration, and apoptosis of SW872 cells in vitro. GPS2 knockdown cell line was established by transfecting SW872 cells with GPS2-shRNA. qPCR and Western blot analysis confirmed the knockdown efficacy of both GPS2-shRNA1 and GPS2-shRNA2 (Fig. 2a, b). Proliferation index showed that transduction of both GPS2-shRNA1 and GPS2-shRNA2 leads to enhanced proliferation of SW872 cells (Fig. 2c, d). This is further confirmed by a BrdU staining assay (Fig. 2e). In concert, elevated glucose consumption was observed in GPS2 knocked-down cell cultures in contrast to the control cells (Fig. 2f). These results suggested that GPS2 may act as a negative regulator of cell proliferation in vitro.

### GPS2 knockdown promoted cell cycle progression with few influences on apoptosis of SW872 cells

**Fig. 3:**
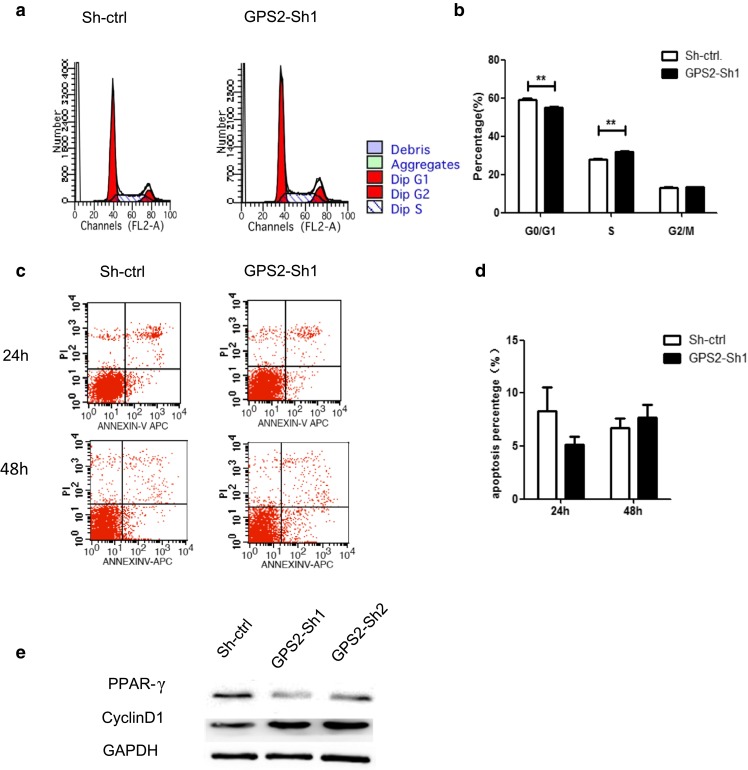
Effects of GPS2 on cell-cycle progression and apoptosis of SW872 cells. **a**, **b** The cells infected with sh-ctrl and GPS2-sh1 were stained with PI, and DNA content was analyzed by flow cytometry. The cell cycle distribution was calculated using ModFit software. **a** The representative plot of DNA content using flow cytometry. **b** The percentage of cell cycle distribution of SW872 cells infected with sh-ctrl or GPS2-sh1 vector. **c**, **d** The SW872 cells transfected with sh-ctrl or GPS2-sh1 vector were in serum-starved culture for 24 or 48 h. Apoptosis was detected by Annexin V-FITC/PI staining. **c** The representative plot of apoptosis assay using flow cytometry. **d** The percentage of apoptotic cells. **e** SW872 cells were transfected with sh-ctrl, GPS2-sh1, and GPS2-sh2 vector. The PPARγ and cyclin D1 protein levels were determined by Western blot, GAPDH as a loading control

In previous results, we showed that GPS2 knockdown resulted in enhanced proliferation of SW872 cells. As the increase of cell numbers could be either attributed to more cell divisions, less cell death, or both, we assessed the influences of GPS2 knockdown on cell cycle progression and apoptosis. Flow cytometry analysis showed that GPS2 knockdown induced more cells into cell cycles, revealed by increased percentages in S phase and G2/M phase (Fig. 3a, b). But the Annexin V-positive cells were comparable in GPS2 knockdown cells and control cells (Fig. 3c, d), suggesting few influences of GPS2 on cell death. Cyclin D is a member of the cyclin protein family that is involved in regulating cell cycle progression. The synthesis of cyclin D is initiated during G1 and drives the G1/S phase transition. We further validated that cyclin D1 was increased in GPS2 knockdown cells. Meanwhile, the adipose differentiation maker PPARγ was downregulated (Fig. 3e). These results suggested that GPS2 could promote cell differentiation and inhibit cell division, but had few impacts on cell death.

### GPS2 knockdown enhances migration of SW872 cells

**Fig. 4:**
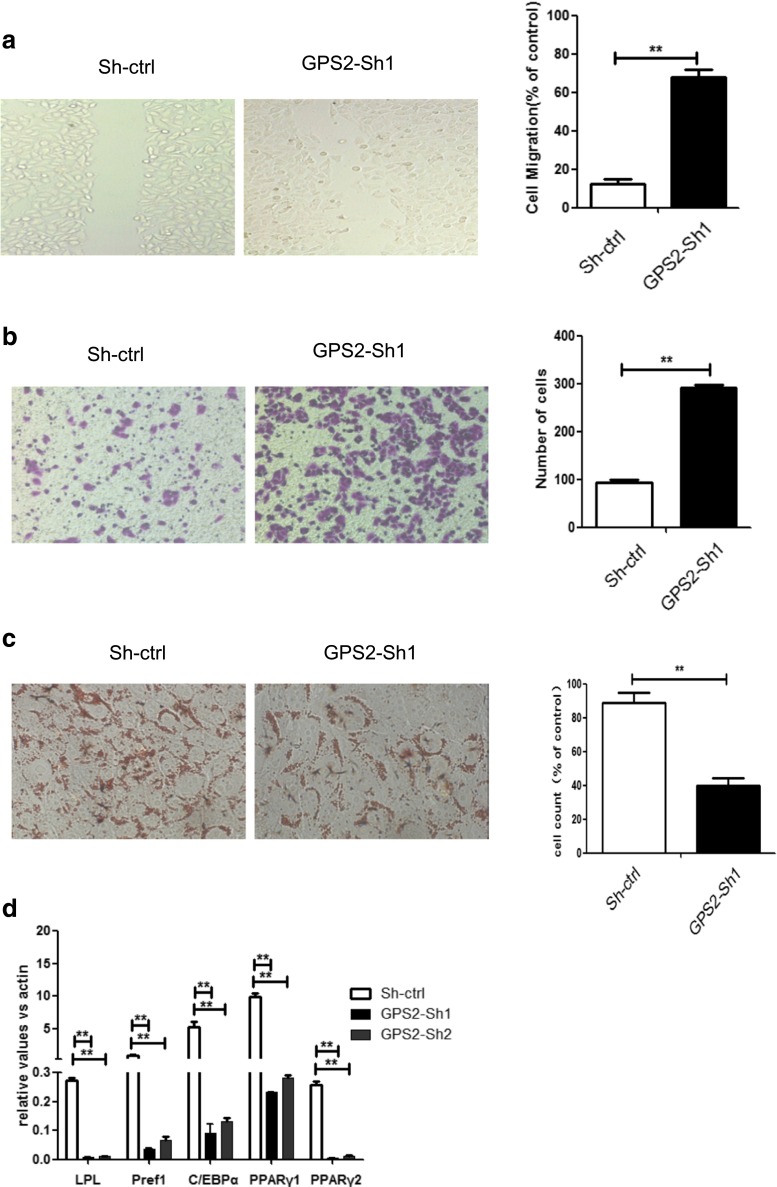
GPS2 knockdown enhances migration and reduces differentiation of SW872 cells. **a**, **b** The SW872 cells transfected with GPS2-sh1 or control vector were evaluated for abilities of migration by using wound-healing assay (the right chart shows the percentage of healing areas) (**a**) and transwell assay( the right chart shows the number of migrated cells ) (**b**). The phenotype experiment showed the ability of adipogenic differentiation after the knockdown of GPS2 ( the right chart shows the percentage of differentiated cells) (**c**). The expression of adipogenic markers, LDL, Pref1, C/EBPα, PPARγ1, and PPARγ2, were determined by Q-PCR in SW872 cells transfected with sh-ctrl, GPS2-sh1, and GPS2-sh2 (**d**)

To validate whether GPS2 regulated cell migration, wound-healing and trans-well assays were performed. The abilities of migration were increased in GPS2 knockdown SW872 cells, in contrast to the control cells (Fig. 4a, b). These results indicated that loss of GPS2 leads to more aggressive phenotypes of SW872 cells. The increased abilities of migration were commonly associated with dedifferentiation, a histologic form of tumor progression, which was also described as epithelial mesenchymal transition (EMT) in its extreme form [23, 24]. GPS2 knockdown also suppressed adipogenic differentiation in SW872 cell line (Fig. 4c). Since GPS2 has been reported to regulate the activities of peroxisome proliferator-activator receptor (PPARγ), the master regulator of adipocyte differentiation [25–27], we checked whether loss of GPS2 resulted in dedifferentiation. As expected, adipose differentiation makers including LPL, Pref1, C/EBP, PPARγ1, and PPARγ2 were decreased in GPS2 knockdown cells than that in control cells (Fig. 4d).

## Discussion

LPS are a group of soft tissue sarcomas with great diversity and low prevalence. Gaining a better understanding of the pathogenesis of these diseases is the first step toward the development of effective targeted therapies to improve outcomes in patients with this disease. However, the molecular pathogenesis of these malignancies are still poorly understood. In this study, we reported that GPS2, a small transactional cofactor, functioned as a tumor suppressor in LPS. More importantly, our data suggested that GPS2 levels were correlated to the prognosis of this disease.

GPS has been shown to regulate suppress G-protein- and RAS- mitogen-activated protein kinase-mediated signal transduction which are required for cell proliferation and migration [15]. By using lentivirus-mediated GPS2 shRNA transduction, we identified that GPS2 silence resulted in enhanced proliferation and migration in SW872 cells in vitro. These data suggested that GPS2 functioned as a tumor suppressor in LPS. Furthermore, we also identified that GPS2 knockdown caused dedifferentiation of these cells. To our knowledge, this is the first report focusing on the biological functions and significance of GPS2 in these malignancies. However, studies with large sample size are still needed to further confirm the importance of GPS2 in LPS.

Besides the genetic amplification of the 12q13-15, other molecular mechanisms of LPS genesis have been proposed in recent years, including deregulation of some miRNAs and their downstream targets, epigenetics, and dysregulation of some oncogenic signaling pathways [4, 10, 28–31]. Particularly, amplification of the oncogene C-JUN is considered to contribute to the inhibition of PPARγ, a key regulator in terminal adipocyte differentiation [32]. In view of that LPS growth was inhibited by downregulating C-JUN via deoxyribozyme (DNAzyme) and that GPS2 was demonstrated to suppress JNK signaling [33], we reasoned that GPS2 may regulate LPS growth and migration through JNK1-PPARγ pathway. This hypothesis will be verified in future studies.

GPS2, first identified as a suppressor of Ras activation, was later shown to be involved in many physiological and pathological processes, including proliferation, apoptosis, DNA repair, brain development, and metabolism [16–20]. The role of GPS2 in adipose differentiation and lipid metabolism has been intensively studied. Toubal et al. reported that SMRT-GPS2 corepressor pathway dysregulation coincides with obesity-linked adipocyte inflammation [22]. GPS2 has been also reported to be required for cholesterol efflux [34]. Together, this body of work implicates that GPS2 was a critical regulator of adipose tissues. However, the role of GPS2 in adipose origined tumors has not been unveiled. The present studies expanded our understanding of the function of GPS2 and highlight the importance of GPS2 in tumorgenesis of soft tissue sarcomas.

In conclusion, our studies identify GPS2 functions as a tumor suppressor in LPS and its downregulation is correlated to prognosis of LPS. These studies enhance our understanding the molecular pathogenesis of LPS and raise the possibility that GPS2 served as a potential prognosis marker.
